# Symbolic ableism in health professions education: development and initial psychometric evaluation of the German symbolic ableism scale

**DOI:** 10.1186/s12909-026-10636-6

**Published:** 2026-10-19

**Authors:** Anna-Lena Hillebrecht, Denise Hausdorf, Rieka von der Warth, Haang Jeung-Maarse, Götz Fabry, Philipp Linde, Marc Auerbacher, Benedikt C. Spies, Daniel R. Reissmann

**Affiliations:** 1 Department of Prosthetic Dentistry, Centre for Dental Medicine, Medical Centre, University of Freiburg, Hugstetter Str. 55, Freiburg, 79106 Germany; 2 Institute of Family Medicine, University Hospital Bonn, University of Bonn, Bonn, Germany; 3 Department of Psychiatry and Psychotherapy, Medical Faculty OWL, Protestant Hospital Bethel Foundation, Bielefeld University, Bielefeld, Germany; 4 Institute for Medical Psychology and Medical Sociology, Faculty of Medicine, University of Freiburg, Freiburg, Germany; 5 Department of Radiation Oncology, CyberKnife and Radiation Therapy, Faculty of Medicine and University Hospital Cologne, University of Cologne, Cologne, Germany; 6 Department of Conservative Dentistry, Periodontology and Digital Dentistry, University Hospital LMU Munich, Munich, Germany; 7 Department of Prosthodontics and Materials Science, Centre for Dental Medicine, University of Leipzig, Leipzig, Germany

**Keywords:** *Prejudice* (MeSH), *Disabled Persons* (MeSH), *Education, Medical* (MeSH), *Education, Dental* (MeSH), *Surveys and Questionnaires* (MeSH)

## Abstract

**Background:**

Ableist attitudes may undermine equity, quality, and safety of care and may also shape the inclusion of learners and professionals with disabilities. Brief, context-sensitive instruments for health professions education are lacking. This study aimed to develop the German-language Symbolic Ableism Scale (SAS-G) and obtain initial exploratory psychometric evidence among medical and dental students.

**Methods:**

A three-phase instrument-development study was conducted between 2022 and 2024. From the outset, people with disabilities participated directly as experts by experience in construct interpretation, item generation, and content review alongside disability-studies and clinical experts. In Phase 1, the original Symbolic Ableism Scale underwent forward-backward translation and was complemented by de novo items generated through this participatory process. Qualitative content analysis and content-validity ratios informed item refinement. In Phase 2, a general-population pilot sample (*N* = 210) completed a 25-item pool; item-level findings guided conceptual and linguistic redevelopment into a 21-item pre-final student version. In Phase 3, 173 medical and dental students completed this version. Factor retention was guided by parallel analysis using Pearson and polychoric correlations and Velicer’s minimum average partial criterion. A primary maximum-likelihood EFA with oblimin rotation was supplemented by an ordinal-data sensitivity analysis using polychoric correlations and MINRES extraction. Internal consistency and hypothesis-generating regressions were examined.

**Results:**

Ten items were retained in two factors: Denial of Rights and Structural Barriers (6 items; Cronbach’s alpha = 0.81; McDonald’s omega = 0.83) and Interpersonal Comfort (4 items; alpha = 0.70; omega = 0.74). The unrotated maximum-likelihood extraction accounted for 44.3% of total item variance, and the ordinal-data sensitivity analysis reproduced all primary item-to-factor assignments. In exploratory regressions, lower Denial of Rights and Structural Barriers scores were associated with female gender, medical study, and friendship with a person with disabilities; lower Interpersonal Comfort scores were associated with medical study and such friendship.

**Conclusions:**

The SAS-G is a brief, participatively developed measure of two complementary dimensions of symbolic ableism in health professions education. Findings provide initial exploratory support for its structure and internal consistency in a single-site student sample; independent multisite confirmation and evidence of temporal stability, responsiveness, and measurement invariance are required before the scale is used to evaluate interventions or compare groups or countries.

**Supplementary Information:**

The online version contains supplementary material available at https://doi.org/10.1186/s12909-026-10636-6.

## Background

Ableism, defined as discriminatory attitudes, assumptions, and practices toward people with disabilities, remains an underrecognized barrier to equitable health care and a formative influence on professional identity development in health professions education [1, 2]. While overt ableism involves explicit devaluation or exclusion of people with disabilities, symbolic ableism operates through subtle belief systems that frame inequality as fair, inevitable, or professionally justified [1].

In medical and dental education, symbolic ableism may be expressed through claims that formally identical treatment, merit-based selection, or conventional notions of professionalism are inherently neutral. Such claims can obscure structural barriers faced by patients, learners, and colleagues with disabilities and shift responsibility for disadvantage from institutions to individuals [3–6]. In the present study, the primary focus is on students’ attitudes toward people with disabilities and their potential relevance to future clinical communication and care; the same beliefs may also contribute to exclusionary learning and working environments for trainees and professionals with disabilities. Symbolic ableism includes denial of continuing discrimination, individualization of inequity, and resistance to accommodations perceived as compromising standards [1, 5–8]. 

International frameworks such as the United Nations Convention on the Rights of Persons with Disabilities (CRPD) [6] call for learning environments that promote disability competence and actively challenge discriminatory norms in health professions education. Despite these commitments, medical and dental curricula continue to offer limited structured exposure to disability-related content. Learners frequently report uncertainty, discomfort, or avoidance in encounters with people with disabilities, particularly in clinical settings. Research consistently demonstrates that both explicit and implicit disability-related bias are prevalent among health professions trainees, underscoring the need for systematic approaches to identifying and addressing attitudinal barriers [9–24].

Despite growing recognition of disability as a core dimension of equity in health professions education, no brief instrument with published psychometric evidence is currently available to assess symbolic ableism in German medical and dental education. The US Symbolic Ableism Scale provides an important foundation, but its Slovenian and Japanese adaptations yielded different structures, underscoring the need to examine rather than assume cross-cultural equivalence. [16, 25, 26] This measurement gap limits rigorous descriptive study of subtle disability-related attitudes; evaluation of change or intervention effects will additionally require evidence of temporal stability and responsiveness.

Cross-cultural adaptation is needed because disability-related instruments do not only translate words, but also assumptions about disability, rights, support, accommodation, and professional responsibility. This is consistent with the World Health Organization’s International Classification of Functioning, Disability and Health (ICF), which conceptualizes functioning and disability as arising in interaction with contextual, including environmental, factors. In the context of symbolic ableism, differences between the United States and Germany may shape how items referring to individual responsibility, fairness, rights, inclusion, or accommodation are understood. These differences include distinct legal and welfare frameworks, educational and employment structures, access pathways within health care, and norms governing clinician-patient communication. Similarly, a recent international consensus study of the International Association for Disability and Oral Health (iADH) Universal Case Mix Tool identified cultural acceptability and differences in legal and service contexts as relevant design considerations for disability-related oral health measurement [27].

Direct involvement of people with disabilities is especially important when defining a construct that concerns their social position and lived experience. The Convention on the Rights of Persons with Disabilities calls for close consultation and active involvement of people with disabilities in decisions concerning them, and General Comment No. 7 emphasizes meaningful participation [6, 28]. Scale-development guidance also recommends combining deductive and inductive item generation, including interviews, with expert review of content relevance and representativeness; GRIPP2 supports transparent reporting of public involvement [29–31]. These principles informed the participatory elements of the present study.

To address this need, the present study developed the German Symbolic Ableism Scale (SAS-G) and examined its dimensionality and internal consistency in an exploratory, single-site student sample. It also explored demographic and contact-related correlates of the resulting subscale mean scores.

## Methods

### Design, ethics, and registration

**Fig. 1:**
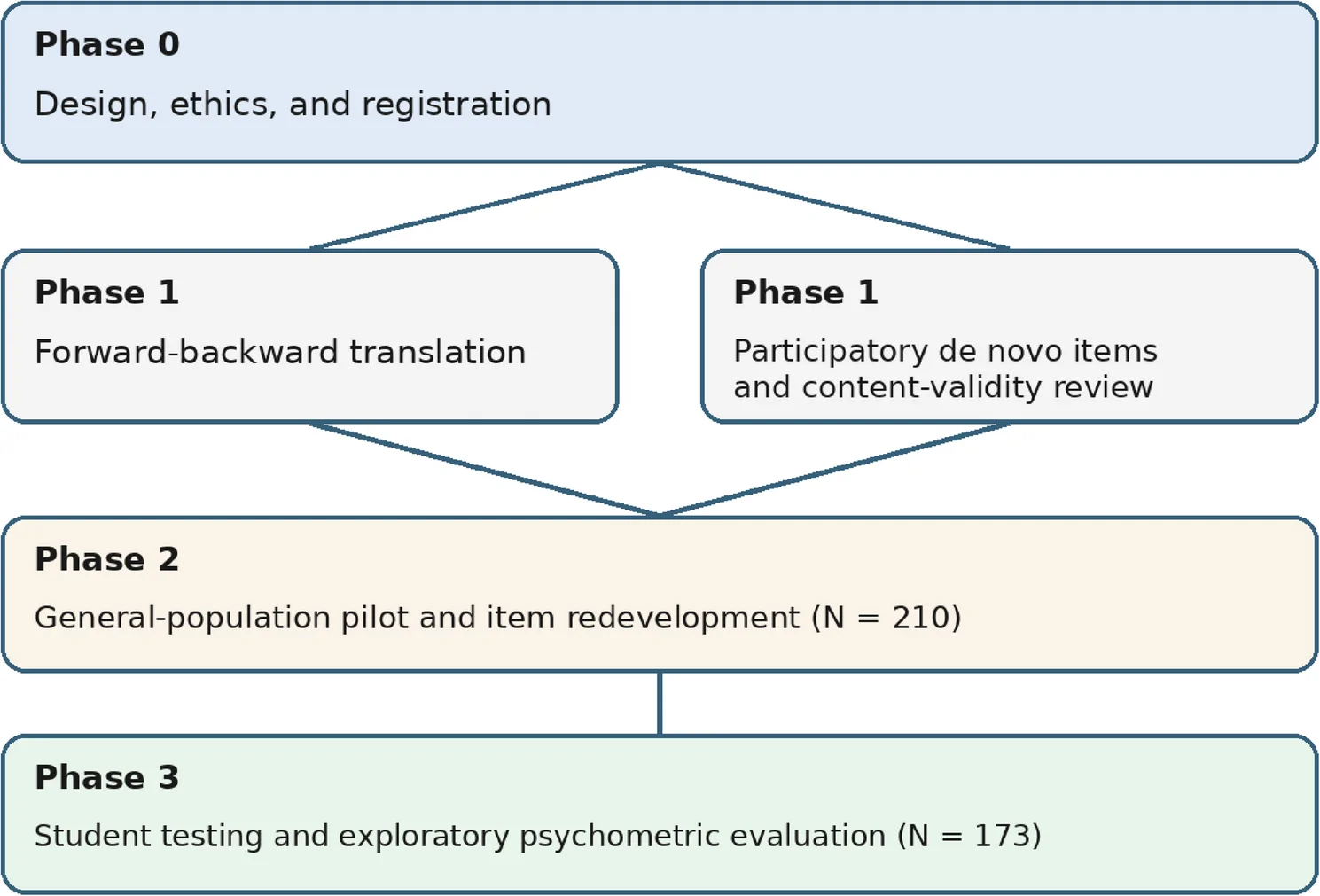
Overview of the three-phase instrument-development process. CVR, content validity ratio

A three-phase instrument-development study with participatory elements was conducted at the University of Freiburg between 2022 and 2024 (Fig. 1). People with disabilities were involved directly from Phase 1—before de novo item generation—as experts by experience in construct interpretation, item generation, and content review. Ethics approval (No. 22-1492-S2; December 13, 2022) and prospective registration (DRKS00031466) were obtained. All Phase-1 activities commenced after ethics approval had been granted on December 13, 2022. Participation was voluntary. For all online components, participants received the study information electronically and provided electronic informed consent by actively selecting a mandatory consent option before participation. The Declaration of Helsinki statement is reported under Ethics approval and consent to participate in the Declarations Sect [32]. 

### Phase 1: Development of pilot version

#### Translation

The original English Symbolic Ableism Scale (SAS) [16] was translated into German using a standardized forward–backward procedure. Two native German speakers fluent in English independently produced forward translations. Discrepancies were resolved by consensus. Three bilingual experts (medicine, dentistry, disability studies) back-translated the reconciled German version into English. Back-translations were compared item-by-item with the source instrument to resolve semantic, idiomatic, and conceptual discrepancies. Emphasis was placed on preserving construct meaning rather than literal wording.

#### De-novo development

To ensure cultural and contextual adequacy for German academic medicine and dentistry, the translated pool was complemented by items generated through a participatory process. The expert panel (*n* = 15; multiple roles possible) included people with physical (*n* = 3), cognitive, sensory, or psychiatric disabilities (*n* = 4); disability-studies scholars (*n* = 3); health professionals (medicine *n* = 2; dentistry *n* = 2); caregivers or support staff (*n* = 2); and family members of people with disabilities (*n* = 4). Thus, lived experience was treated as a source of construct knowledge from the outset rather than sought only as a later acceptability check. [6, 28, 30, 31] Guided online interviews and structured email prompts explored established dimensions of symbolic ableism as well as themes salient in the German context. Structured qualitative content analysis mapped responses onto individualism, recognition of continuing discrimination, empathy or affect, and perceived societal burden, while also identifying context-specific themes: perceived adequacy of health care, societal and labour-market representation, contact avoidance, and infantilizing communication. Compared with the original US scale’s emphasis on merit, individual responsibility, and denial of discrimination, these additions extended coverage toward institutional access and everyday clinical interaction. The interview guide is provided in Supplementary Table 9 [33]. 

#### Evaluation of pilot version

Panel members completed a REDCap-based content-validity survey and provided item-level comments on clarity and relevance. Content Validity Ratios (CVRs) were calculated according to Lawshe’s method, with critical-value guidance from Ayre and Scally [34]. A study threshold of CVR ≥ 0.60 was used to identify items requiring revision rather than as an automatic exclusion rule. Items below this threshold were revised using the qualitative comments (e.g., simplification, avoidance of double-barrelled wording, and register alignment for student populations) and retained for pilot testing. The resulting wording constituted the Phase-2 test form.

### Phase 2: Pre-testing of pilot version

#### Study population

A German general-population sample was recruited via an online panel to ensure sociocultural heterogeneity. This sample was used for item stress-testing and linguistic refinement rather than for establishing the final factor structure, because the aim of Phase 2 was to examine whether item wording and response patterns were sufficiently variable and interpretable across a heterogeneous German-speaking population before testing the pre-final student version. Of 313 individuals who accessed the survey, 210 provided complete data and were included in the analyses.

#### Pilot version

The pilot version consisted of 12 de-novo items and 13 translated SAS items, resulting in a preliminary 25-item pool. Responses could be made on a 5-point Likert scale (1 = strongly disagree to 5 = strongly agree), including reverse-scored items.

#### Item analysis

Items were flagged for missingness > 5%, absolute skew ≥ 2, kurtosis ≥ 7, and floor/ceiling effects > 50%. Items with pronounced floor/ceiling effects were removed.

#### Statistical analysis

Phase 2 was an item-development step rather than a confirmatory psychometric test. Preliminary item-level and multivariate analyses were used only to identify redundancy, extreme distributions, and conceptually problematic response patterns in the 25-item pool. Because these analyses did not determine the structure tested in Phase 3, their description and results have been intentionally shortened.

#### Revision of pilot version

Based on the Phase-2 findings, no wording was carried forward unchanged by default. Concepts were retained, consolidated, reframed, or newly introduced according to four criteria: removal of redundancy, avoidance of extreme or double-barrelled wording, use of a consistent first-person perspective, and clearer representation of cognitive and interpersonal facets of symbolic ableism. Supplementary Tables 3 A and 3B map the 25 Phase-2 items to the 21-item pre-final version and document the type and rationale of each change.

Affective and interpersonal content was strengthened, and wording was shifted to a first-person perspective. The resulting 21-item pool was informed by the theoretical framework, qualitative findings, Phase-2 response patterns, and established approaches to attitude-scale development. [29, 30]

### Phase 3: Testing the pre-final version and initial psychometric evaluation

#### Population

Medical and dental students at the University of Freiburg were invited via institutional mailing lists and snowballing. Eligibility criteria were age ≥ 18 years, current enrollment, and provision of informed consent. Of 180 who accessed the survey, *N* = 173 provided complete data.

#### Pre-final version

The 25-item Phase-2 pool was conceptually and linguistically redeveloped into a 21-item pre-final version before student testing. This transition was not a one-to-one translation: some concepts were consolidated or reframed, all literal Phase-2 wordings were retired, and ten items were newly introduced to extend affective, interpersonal, and stereotype content (Supplementary Tables 3 A and 3B).

The pre-final pool covered cognitive (rights, competence, and structural fairness) and interpersonal facets of symbolic ableism. The study questionnaire had not been published previously in this form. The supplementary material provides the final German 10-item target-language instrument (Supplementary Table 6), English-language renderings of the 21-item pre-final pool and the final 10-item SAS-G (Supplementary Tables 7 and 8), and the conceptual mapping from Phase 2 to Phase 3 (Supplementary Tables 3 A and 3B). Responses used a five-point Likert scale (1 = strongly disagree to 5 = strongly agree). Positively worded items were reverse-scored, and each final subscale score was calculated as the arithmetic mean of its constituent items. Higher item and subscale mean scores therefore indicated more pronounced ableist attitudes; specifically, higher Denial of Rights and Structural Barriers scores indicate stronger denial or minimization of rights, discrimination, and structural barriers, and higher Interpersonal Comfort scores indicate greater discomfort, avoidance, or infantilizing communication.

#### Item and statistical analysis

The number of factors was determined by parallel analysis based on both Pearson and polychoric correlation matrices, complemented by Velicer’s MAP criterion. The primary EFA used a Pearson correlation matrix, maximum-likelihood extraction, and oblimin rotation (delta = 0). The prespecified Pearson/maximum-likelihood analysis was retained for continuity with the initial analytic plan and because items remaining after distributional screening did not show extreme endpoint clustering.

Sampling adequacy was evaluated using the KMO statistic and Bartlett’s test of sphericity. Items with primary loadings below 0.32 or salient cross-loadings were considered for removal, with conceptual coverage reviewed alongside statistical criteria. Given the five-category ordinal response format, robustness was examined in a sensitivity EFA using a polychoric correlation matrix, MINRES extraction, and oblimin rotation. Internal consistency was assessed with Cronbach’s alpha, McDonald’s omega, corrected item-total correlations, and alpha if each item was deleted. Analyses were performed in SPSS version 27.0 (IBM Corp.) and R version 4.2.1, using the psych and polycor packages.

Hypothesis-generating analyses examined bivariate associations between subscale mean scores and demographic and contact variables. Variables significant in bivariate tests were entered into separate multiple linear regression models for each subscale. Coding was age in years; gender 0 = female and 1 = male; study programme 0 = dentistry and 1 = medicine; and friendship with a person with disabilities 0 = yes and 1 = no. Because higher subscale scores indicated more ableism, a positive coefficient for gender represented higher scores among men, a negative coefficient for study programme represented lower scores among medical students, and a positive coefficient for friendship coding represented higher scores among students without such friendships. Model assumptions were checked (variance inflation factors < 5; Durbin-Watson 1.5–2.5; standardized residual diagnostics; normal P-P plots). Missing data were handled by listwise deletion. No adjustment for multiple testing was applied; all association analyses are therefore hypothesis-generating.

## Results

### Phase 1: Development of pilot version

The forward–backward translation process produced a German version that was semantically and conceptually equivalent to the English SAS. Minor discrepancies (e.g., idiomatic differences) were resolved in consensus conferences; no items were eliminated at this stage.

The participatory panel, including people with disabilities as experts by experience, generated 12 additional items addressing contextual dimensions specific to German academic medicine and dentistry (e.g., infantilization in clinical encounters and avoidance of contact in student-patient interactions). Together with the 13 translated items, this produced a preliminary 25-item pool. Most items met the study’s CVR threshold (≥ 0.60); items below the threshold were revised using qualitative feedback and retained for pilot testing.

### Phase 2: Pre-testing of pilot version

#### Study population

A total of 210 individuals from the German general population completed the pilot survey in Phase 2. The mean age of participants was 41.6 years (SD = 13.8), with 120 (57.1%) identifying as female, 89 (42.4%) as male, and 1 (0.5%) as diverse. Regarding personal impairments, 26 (12.4%) reported a physical impairment, 6 (2.9%) a sensory impairment, 13 (6.2%) a mental or emotional impairment, and 3 (1.4%) a learning impairment, while 168 (80.0%) indicated having no impairments.

Social contact with people with disabilities was common: 105 participants (50.0%) reported contact within their circle of friends, 104 (49.5%) within their family, and 202 (96.2%) in daily life. Five participants (2.4%) reported consciously avoiding contact. Detailed Phase-2 sample characteristics are provided in Supplementary Table 1.

#### Item analysis

Mean item scores in the Phase-2 pilot (*N* = 210) ranged from 1.1 to 3.2 on the five-point Likert scale (Supplementary Table 2). Five items (7, 8, 9, 10, and 16) showed floor effects of at least 50% and were excluded from the provisional exploration. Analyses of the remaining items revealed redundancy and response patterns that did not support a substantively defensible score interpretation. Because these analyses served only to trigger redevelopment and did not determine the structure tested in Phase 3, no Phase-2 factor solution is presented as validation evidence. The findings motivated a conceptual and linguistic overhaul rather than incremental trimming.

#### Factor structure

Preliminary modelling did not yield a score interpretation suitable for carry-forward. In particular, some respondents obtained identical total scores despite differing on the item representing active rejection. Thus, a single aggregate score could mask substantively distinct response profiles, and several items also behaved counter to theoretical expectations. The Phase-2 model was therefore not treated as validation evidence and did not determine the factor structure examined in Phase 3.

#### Revised instrument

Five of the 25 items were removed during initial Phase-2 screening, but the remaining items were not treated as a candidate final scale. Because the preliminary patterns were conceptually incoherent, the team redeveloped the content rather than incrementally trimming the original pool. Supplementary Tables 3 A and 3B distinguish concepts that were consolidated or reframed from content that was newly introduced.

The resulting 21-item version was designed to capture cognitive and interpersonal dimensions of symbolic ableism in German medical and dental education (Supplementary Tables 3 A and 3B).

### Phase 3: Pre-final version

#### Study population

A total of 173 students provided complete Phase-3 data: 91 (52.6%) medical and 82 (47.4%) dental students (Table 1). Mean age was 23.2 years (SD = 3.3); 135 (78.0%) participants identified as female and 38 (22.0%) as male. Participants were enrolled in semesters 1 through 11; the semester-specific distribution is reported in Table 1. Twelve (6.9%) reported a personal impairment. Contact with people with disabilities was reported within the family by 67 (38.7%), within the circle of friends by 59 (34.1%), during studies by 45 (26.0%), and in daily life by 157 (90.8%); four (2.3%) reported conscious avoidance.

**Table 1:** Sociodemographic characteristics of the phase-3 student sample

		Mean (SD) or *n* (%)
Age (years)		23.2 (3.3)
Gender	female	135 (78.0)
	male	38 (22.0)
Study programme	dentistry	82 (47.4)
	medicine	91 (52.6)
Semester	1st semester	41 (23.7)
	2nd semester	9 (5.2)
	3rd semester	14 (8.1)
	4th semester	21 (12.1)
	5th semester	12 (6.9)
	6th semester	9 (5.2)
	7th semester	14 (8.1)
	8th semester	6 (3.5)
	9th semester	14 (8.1)
	10th semester	12 (6.9)
	11th semester	21 (12.1)
Personal impairments (multiple answers possible)	physical impairment	4 (2.3)
	sensory impairment	2 (1.2)
	mental / emotional impairment	5 (2.9)
	learning impairment	1 (0.6)
	no impairments	161 (93.1)
Contact in the circle of friends	yes	59 (34.1)
Contact in family	yes	67 (38.7)
Contact in studies	yes	45 (26.0)
	no	50 (28.9)
	I don’t know	78 (45.1)
Contact in daily life	yes	157 (90.8)
Conscious avoidance	yes	4 (2.3)

*Abbreviations*: *SD *standard deviation

#### Item-level analyses

The pre-final instrument comprised 21 items. Seven items (7, 8, 13, 14, 15, 17, and 18) exceeded the prespecified endpoint-concentration threshold (> 50%) and were excluded before factor analysis. Descriptive statistics for all 21 items are provided in Supplementary Table 5; the item-specific endpoint percentages and reasons for all exclusions are reported in Supplementary Table 10.

The remaining 14 items demonstrated acceptable distributions without excessive response clustering. Mean item scores ranged from 1.7 to 3.3 (Supplementary Table 4), indicating sufficient variability for factor analytic procedures.

#### Factor structure

The maximum-likelihood EFA with oblimin rotation yielded a two-factor solution with 10 final items. Sampling adequacy statistics for this final 10-item set were KMO = 0.796 and Bartlett’s test chi-square = 523.1 (*p* < .001). Parallel analyses based on Pearson and polychoric correlations supported two factors. For the Pearson/maximum-likelihood analysis, 100 random datasets were simulated, and the 95th percentile of the simulated eigenvalues was used as the retention criterion. The observed eigenvalues for Factors 1, 2, and 3 were 3.357, 1.092, and 0.162, respectively, compared with 95th-percentile simulated eigenvalues of 1.116, 0.397, and 0.301. For the polychoric/MINRES sensitivity analysis, 20 random datasets were simulated, yielding observed eigenvalues of 4.139, 1.306, and 0.177 for Factors 1, 2, and 3, compared with 95th-percentile simulated eigenvalues of 1.081, 0.397, and 0.262, respectively. Velicer’s MAP criterion likewise supported a two-factor solution, with the minimum MAP value at two factors (MAP = 0.031).

During iterative EFA, four additional items were removed. Items 3 and 5 loaded on a separate third factor (loadings 0.555 and 0.524, respectively), which was not retained because it contained only those two items. Item 4 had no loading of at least 0.32, and Item 12 cross-loaded (primary 0.442; secondary 0.326). The remaining 10 items loaded on two interpretable factors: Denial of Rights and Structural Barriers (six items) and Interpersonal Comfort (four items). The unrotated maximum-likelihood extraction accounted for 44.3% of total item variance (30.7% and 13.6% before rotation). Because oblimin rotation permits correlated factors, the rotated factor-specific sums are not additive (Table 2; Supplementary Table 10).

**Table 2:** Pattern coefficients from the primary maximum-likelihood EFA for the final 10-item SAS-G (Factor 1, Denial of rights and structural barriers; Factor 2, Interpersonal comfort)

Item (long form)	Factor 1	Factor 2
1. I am often unsure how to behave when I interact with people with disabilities		.71
2. I often feel uneasy when talking to people with disabilities		.80
6. I tend to avoid contact with people with disabilities		.58
9. I believe that discrimination against people with disabilities is no longer a problem in Germany	.80	
10. I think people with disabilities are less competent in many areas	.42	
11. I think social structures make life more difficult for people with disabilities ^r^	.49	
16. I consider medical care for people with disabilities to be adequate	.68	
19. I think people with disabilities are already sufficiently represented in all areas of society	.76	
20. I think people with disabilities should have greater access to the regular labor market ^r^	.66	
21. I tend to use a childish, caring tone when talking to people with disabilities		.36

Response options: 1 = strongly disagree to 5 = strongly agree. Higher subscale means indicate more ableist attitudes

Superscript r indicates reverse-scored items

The ordinal-data sensitivity EFA using polychoric correlations, MINRES extraction, and oblimin rotation reproduced the two-factor structure and every primary item-to-factor assignment. Loadings ranged from 0.44 to 0.87 for Factor 1 and from 0.42 to 0.88 for Factor 2, and the factors correlated at *r* = .31. This convergence supports the robustness of the exploratory item assignment to the correlation matrix and extraction method.

#### Scale statistics

For Denial of Rights and Structural Barriers (6 items), Cronbach’s alpha was 0.81 and McDonald’s omega was 0.83; corrected item-total correlations ranged from 0.43 to 0.64, and alpha if an item was deleted ranged from 0.75 to 0.80. For Interpersonal Comfort (4 items), alpha was 0.70 and omega was 0.74; corrected item-total correlations ranged from 0.29 to 0.62, and alpha if an item was deleted ranged from 0.53 to 0.75. Item 21 had the lowest corrected item-total correlation (*r* = .29), and alpha would increase to 0.75 if it were deleted. It was retained because its loading exceeded the prespecified threshold and its content—infantilizing communication—was judged conceptually important. Reliability of the four-item factor should therefore be regarded as acceptable but limited (Supplementary Table 11).

#### Associated factors

In the model for higher Denial of Rights and Structural Barriers, men had higher scores than women (beta = 0.25, *p* < .001), whereas medical students had lower scores than dental students (beta = − 0.40, *p* < .001). Students without a friend with disabilities had higher scores than those reporting such a friendship (beta = 0.15, *p* = .034). The model explained 24% of adjusted variance (adjusted R-squared = 0.24). For Interpersonal Comfort, medical students had lower scores than dental students (beta = − 0.16, *p* = .038), and students without a friend with disabilities had higher scores than those reporting such a friendship (beta = 0.22, *p* = .005); adjusted explained variance was 7%. These analyses are exploratory and should not be interpreted causally (Table 3).

**Table 3:** Multiple linear regression models for phase-3 subscale mean scores

	Factor 1: Denial of Rights and Structural Barriers				Factor 2: Interpersonal Comfort			
	B (SE)	95% CI	ß	*p*	B (SE)	95% CI	ß	*p*
Age	.002 (0.011)	[-.019, .023]	.011	.87	− .009 (0.015)	[-.038, 0.020]	− .046	.54
Gender	.325 (0.086)	[.154, .495]	.251	< .001	.080 (0.118)	[-.152, .313]	.051	.50
Study programme	− .433 (.073)	[-.576, − .289]	− .403	< .001	− .207 (0.099)	[-.402, − .011]	− .157	.04
Contact in the circle of friends	.164 (0.077)	[.013, .316]	.145	.03	.300 (0.104)	[.094, .506]	.216	.005
R^2^ / adjusted R^2^	.26/.24				.09/.07			

Statistical significance was set at *p* < .05. Coding: gender 0 = female, 1 = male; study programme 0 = dentistry, 1 = medicine; friendship with a person with disabilities 0 = yes, 1 = no. Outcomes were arithmetic subscale mean scores after reverse scoring; higher scores indicate more ableist attitudes

*B *unstandardized coefficient, *SE *standard error, *CI *confidence interval, *beta *standardized coefficient

## Discussion

This study developed the 10-item SAS-G and obtained initial exploratory evidence of a two-factor structure and internal consistency in German medical and dental students. The findings should be regarded as scale-development evidence rather than independent confirmation of validity.

The identified dimensions, Denial of Rights and Structural Barriers and Interpersonal Comfort, capture complementary components of symbolic ableism: cognitive judgments about entitlement, competence, and structural fairness, and interaction-related tendencies such as uncertainty, discomfort, and avoidance. Together, they provide an empirically grounded structure for assessing subtle ableist attitudes that may influence clinical communication and decision-making, even in the absence of overt prejudice. This distinction is educationally consequential: interaction-related attitudes are rarely addressed explicitly in formal curricula yet are powerfully shaped through the hidden curriculum and clinical role modeling [35–37].

The higher Denial of Rights and Structural Barriers factor captures beliefs about recognition or denial of discrimination, competence, representation, health-care adequacy, labour-market inclusion, and structural barriers affecting people with disabilities. These beliefs represent central elements of symbolic ableism: discrimination may be minimized, and inequalities may be attributed to individual rather than social factors. [16, 38, 39] The Interpersonal Comfort factor captures relational processes such as uncertainty, avoidance, and infantilizing communication patterns, which are particularly relevant in clinical encounters and may shape patient-provider interaction. Together, these dimensions illustrate how symbolic ableism operates through both value-based judgments and everyday interactional tendencies, thereby potentially influencing how learners perceive and engage with patients with disabilities even in the absence of overt prejudice [40–43]. 

Lower Denial of Rights and Structural Barriers scores were associated with female gender, medical study, and friendship with a person with disabilities; lower Interpersonal Comfort scores were associated with medical study and such friendship [44–48]. The friendship finding is consistent with intergroup-contact research, whereas the programme difference may reflect curricular exposure, self-selection, or unmeasured cohort characteristics. [9, 15, 16, 19, 49–56] Because the sample was single-site and predominantly female, the regressions were cross-sectional, and no correction for multiple testing was applied, these findings should be treated as hypothesis-generating rather than causal or generalizable.

Reliance on self-report raises the possibility of social-desirability effects, particularly among students socialized toward professional norms. The seven endpoint-concentrated items may have been strongly rejected because of such norms rather than because their content was irrelevant. Removing them improved variability for factor analysis but may have narrowed content coverage, especially for overtly exclusionary or normatively proscribed beliefs. This trade-off should be examined in future studies using social-desirability measures, cognitive interviewing, and alternative response formats.

Attitudinal bias is a relevant but incompletely measured dimension of health-professions learning environments. At its present stage, the SAS-G may be used cautiously for descriptive research and hypothesis generation. It should not yet be used to infer attitudinal change or intervention effectiveness, because test-retest reliability, responsiveness, and minimally important change have not been established.

In disability-related oral health care, attitudes should also be distinguished from clinical case complexity and objectively assessed adaptation needs. The iADH Universal Case Mix Tool describes seven adaptation domains; communication, dental behaviour support, medical status, risk factors for oral disease and dysfunction, autonomy, legal and ethical barriers, and access to adapted care at service, system, and policy levels [27]. Conceptually, future studies could test whether SAS-G scores are associated with learners’ recognition of such needs; the present study did not examine that relationship.

The two-factor solution differs from the original US SAS and from Slovenian and Japanese adaptations. [16, 25, 26] Variation across versions may reflect differences in language, social policy, disability rights, educational exposure, and response norms. This supports culturally grounded development but limits direct score comparison. International comparisons will require harmonized content and formal tests of configural, metric, and scalar measurement invariance rather than reliance on similarly labelled factors.

A major strength is that people with disabilities were directly involved from the beginning of construct interpretation and de novo item generation through content review, alongside disability-studies and clinical-education expertise. Lived experience therefore informed what was measured rather than serving only as a later acceptability check, consistent with disability-participation principles and scale-development guidance. [6, 28, 30, 31] This approach strengthened content relevance and reduced the risk of reproducing exclusively professional assumptions, but it does not substitute for independent psychometric confirmation. Important limitations remain. The Phase-3 sample was modest, drawn from one institution, and predominantly female; these characteristics may influence both the exploratory factor solution and regression estimates. The final structure was derived and evaluated in the same sample, no CFA was conducted in an independent cohort, and sparse categories prevented robust analysis of participants with disabilities and some contact variables. Self-report may underestimate socially undesirable attitudes. Structural and institutional ableism were outside the scale’s scope, and evidence for test-retest reliability, responsiveness, criterion validity, and measurement invariance is not yet available.

Future research should first confirm the two-factor structure using CFA in a larger, independent, multisite, and more gender-diverse sample. It should then examine test-retest reliability, convergent and discriminant validity, responsiveness to educational interventions, and measurement invariance across programmes, training stages, disability status, gender, institutions, languages, and countries. Only after such evidence is available should the scale be used for longitudinal evaluation, intervention-effect estimation, or international score comparisons.

## Conclusion

The SAS-G provides a brief, participatively developed measure of two complementary dimensions of symbolic ableism in German health professions education: denial of rights and structural barriers, and interpersonal discomfort. In this single-site student sample, converging exploratory analyses supported its two-factor structure. Internal consistency was good for Denial of Rights and Structural Barriers and acceptable but limited for the four-item Interpersonal Comfort subscale. The SAS-G may therefore support cautious descriptive and hypothesis-generating research, but independent multisite confirmation and evidence of temporal stability, responsiveness, and measurement invariance are required before it is used to evaluate interventions or compare groups, languages, or countries.

## Supplementary Information

Supplementary Material 1.

## Data Availability

The datasets generated and/or analysed during the current study are available from the corresponding author on reasonable request. English-language versions of the study questionnaire and interview guide are provided in the supplementary materials.

## Abbreviations

SAS-G: German symbolic ableism scale
SAS: Symbolic ableism scale
CRPD: Convention on the rights of persons with disabilities
EFA: Exploratory factor analysis
MAP: Minimum average partial
MINRES: Minimum residual
KMO: Kaiser-Meyer-Olkin statistic
CVR: Content validity ratio
SD: Standard deviation
CI: Confidence interval
