# Supplementary material for: Symbolic ableism in health professions education: development and initial psychometric evaluation of the German symbolic ableism scale
Source: BMC Med Educ. 2026 Oct 19;26:1681. doi: 10.1186/s12909-026-10636-6 (PMC13681877; doi:10.1186/s12909-026-10636-6)
Supplement: Supplementary file 1 [file 12909_2026_10636_MOESM1_ESM.docx]

***Supplementary Material***

*Symbolic ableism in health professions education: Development and initial psychometric evaluation of the German Symbolic Ableism Scale*

**Supplementary Table 1. Sociodemographic characteristics of the Phase-2 general-population sample**

| **Characteristic** | **Category** | **Mean (SD) or n (%)** |
| --- | --- | --- |
| Age (years) |  | 41.6 (13.8) |
| Gender | female | 120 (57.1) |
|  | male | 89 (42.4) |
|  | diverse | 1 (0.5) |
| Personal impairments (multiple answers possible) | physical impairment | 26 (12.4) |
|  | sensory impairment | 6 (2.9) |
|  | mental / emotional impairment | 13 (6.2) |
|  | learning impairment | 3 (1.4) |
|  | no impairments | 168 (80.0) |
| Contact in the circle of friends | yes | 105 (50.0) |
| Contact in family | yes | 104 (49.5) |
| Contact in daily life | yes | 202 (96.2) |
| Conscious avoidance | yes | 5 (2.4) |

**Note.** SD, standard deviation.

**Supplementary Table 2. Descriptive statistics of the 25 preliminary SAS-G items in the Phase-2 general-population pilot (N = 210)**

| **Item** | **Item wording** | **Mean (SD)** | **Range** | **Floor (%)** | **Ceiling (%)** |
| --- | --- | --- | --- | --- | --- |
| 1 | Even if disabled people try hard they often cannot reach their goals^r^. | 3.0 (1.2) | 1-5 | 6.2 | 11.4 |
| 2 | Even if disabled people are ambitious they often cannot succeed^r^. | 3.2 (1.3) | 1-5 | 6.2 | 16.7 |
| 3 | If disabled people work hard they almost always get what they want. | 2.6 (0.9) | 1-5 | 8.6 | 1.9 |
| 4 | Hard work offers little guarantee of success for disabled people^r^. | 3.1 (1.0) | 1-5 | 3.3 | 5.2 |
| 5 | Any disabled person who is willing to work hard has a good chance of succeeding. | 2.7 (1.0) | 1-5 | 9.0 | 1.9 |
| 6 | Discrimination against disabled people is no longer a problem in Germany. | 1.7 (0.7) | 1-5 | 44.8 | 0.5 |
| 7 | If disabled people would just try harder they would be as well off as nondisabled people. | 1.5 (0.6) | 1-5 | 58.6 | 0.0 |
| 8 | Disabled people are demanding too much from the rest of society. | 1.6 (0.7) | 1-5 | 50.5 | 0.0 |
| 9 | Disabled people should stay hidden. | 1.1 (0.2) | 1-5 | 93.8 | 0.0 |
| 10 | Most disabled people who do not get ahead should not blame the system; they really have only themselves to blame. | 1.5 (0.7) | 1-5 | 60.5 | 0.0 |
| 11 | Over the past few years disabled people have gotten less than they deserve^r^. | 2.3 (0.9) | 1-5 | 19.5 | 1.0 |
| 12 | It is easy to understand the anger of disabled people in Germany^r^. | 2.2 (0.9) | 1-5 | 21.9 | 0.5 |
| 13 | Disabled people complain too much about their situation in society. | 2.0 (0.8) | 1-5 | 30.0 | 0.5 |
| 14 | People with disabilities make an important contribution to diversity in our society^r^. | 1.8 (0.8) | 1-5 | 41.0 | 1.0 |
| 15 | If people with disabilities make an effort, they have the same opportunities to achieve financial independence as people without disabilities. | 2.2 (0.9) | 1-5 | 21.9 | 1.0 |
| 16 | People with disabilities must be given the opportunity to define their own individual goals and to help shape their own living environments^r^. | 1.5 (0.6) | 1-5 | 57.1 | 0.0 |
| 17 | If people with disabilities made more of an effort, they would have the same career and training opportunities as people without disabilities. | 1.8 (0.7) | 1-5 | 34.3 | 0.0 |
| 18 | People with disabilities are generally better supported in sheltered workshops than in the mainstream labour market. | 2.1 (0.9) | 1-5 | 28.1 | 0.5 |
| 19 | People with disabilities are sufficiently represented in all areas of society (for example, on TV, in politics, at events, etc.). | 1.9 (0.7) | 1-5 | 33.8 | 0.0 |
| 20 | People with disabilities receive all the necessary medical care required due to their functional limitations. | 2.5 (1.0) | 1-5 | 19.0 | 1.9 |
| 21 | In recent years, sufficient work has been done to promote equal opportunities for people with disabilities. | 2.1 (0.8) | 1-5 | 20.0 | 0.5 |
| 22 | People with disabilities are to be pitied because of their disability. | 2.1 (0.9) | 1-5 | 31.0 | 0.0 |
| 23 | I show more interest in people without disabilities than in people with disabilities. | 2.4 (0.9) | 1-5 | 17.6 | 0.0 |
| 24 | The provision of specialist staff and support programmes for people with disabilities is currently adequate. | 2.05 (0.8) | 1-5 | 25.7 | 0.5 |
| 25 | A shift in societal attitudes is necessary to ensure that people with disabilities have a comparable level of opportunities^r^. | 1.71 (0.8) | 1-5 | 45.2 | 1.0 |

**Note.** Items with floor or ceiling effects >50% (Items 7, 8, 9, 10, and 16) were excluded from subsequent provisional modelling. ^r^ Reverse-coded. SD, standard deviation.

**Supplementary Table 3A. Exact item inventories and provenance**

| **P2 item** | **Phase-2 wording** | **Phase-2 provenance** | **P2 scoring** | **P3 item** | **Phase-3 wording** | **P3 scoring** |
| --- | --- | --- | --- | --- | --- | --- |
| 1 | Even if disabled people try hard they often cannot reach their goals | Original English SAS | Reverse | 1 | I am often unsure how to behave when I interact with people with disabilities. | Direct |
| 2 | Even if disabled people are ambitious they often cannot succeed | Original English SAS | Reverse | 2 | I often feel uneasy when talking to people with disabilities. | Direct |
| 3 | If disabled people work hard they almost always get what they want | Original English SAS | Direct | 3 | I sometimes feel pity for people with disabilities. | Direct |
| 4 | Hard work offers little guarantee of success for disabled people | Original English SAS | Reverse | 4 | I believe that a disability also determines the character of a person with disabilities. | Direct |
| 5 | Any disabled person who is willing to work hard has a good chance of succeeding | Original English SAS | Direct | 5 | I think people with disabilities, such as dwarfism or Down syndrome, are cute. | Direct |
| 6 | Discrimination against disabled people is no longer a problem in Germany | Original English SAS | Direct | 6 | I tend to avoid contact with people with disabilities. | Direct |
| 7 | If disabled people would just try harder they would be as well off as nondisabled people | Original English SAS | Direct | 7 | I think all people with disabilities should be treated as equals. | Reverse |
| 8 | Disabled people are demanding too much from the rest of society | Original English SAS | Direct | 8 | I think people with disabilities cannot perform well and consequently cannot be successful because of their disability. | Direct |
| 9 | Disabled people should stay hidden | Original English SAS | Direct | 9 | I believe that discrimination against people with disabilities is no longer a problem in Germany. | Direct |
| 10 | Most disabled people who do not get ahead should not blame the system; they really have only themselves to blame | Original English SAS | Direct | 10 | I think people with disabilities are less competent in many areas. | Direct |
| 11 | Over the past few years disabled people have gotten less than they deserve | Original English SAS | Reverse | 11 | I think social structures make life more difficult for people with disabilities. | Reverse |
| 12 | It is easy to understand the anger of disabled people in Germany | Original English SAS | Reverse | 12 | I think it is justified that people with disabilities do not have the same opportunities as people without disabilities because of their disability. | Direct |
| 13 | Disabled people complain too much about their situation in society | Original English SAS | Direct | 13 | I think people with disabilities demand too much from the rest of society. | Direct |
| 14 | People with disabilities make an important contribution to diversity in our society | Participatory de novo item | Reverse | 14 | I think people with disabilities should not hold leadership positions. | Direct |
| 15 | If people with disabilities make an effort, they have the same opportunities to achieve financial independence as people without disabilities | Participatory de novo item | Direct | 15 | I think people with disabilities should stay among themselves. | Direct |
| 16 | People with disabilities must be given the opportunity to define their own individual goals and to help shape their own living environments | Participatory de novo item | Reverse | 16 | I consider medical care for people with disabilities to be adequate. | Direct |
| 17 | If people with disabilities made more of an effort, they would have the same career and training opportunities as people without disabilities | Participatory de novo item | Direct | 17 | I think people with disabilities should have the same political freedoms as people without disabilities. | Reverse |
| 18 | People with disabilities are generally better supported in sheltered workshops than in the mainstream labour market | Participatory de novo item | Direct | 18 | I find it unpleasant to see people with disabilities on television. | Direct |
| 19 | People with disabilities are sufficiently represented in all areas of society (for example, on TV, in politics, at events, etc.) | Participatory de novo item | Direct | 19 | I think people with disabilities are already sufficiently represented in all areas of society. | Direct |
| 20 | People with disabilities receive all the necessary medical care required due to their functional limitations | Participatory de novo item | Direct | 20 | I think people with disabilities should have greater access to the regular labor market. | Reverse |
| 21 | In recent years, sufficient work has been done to promote equal opportunities for people with disabilities | Participatory de novo item | Direct | 21 | I tend to use a childish, caring tone when talking to people with disabilities. | Direct |
| 22 | People with disabilities are to be pitied because of their disability | Participatory de novo item | Direct |  |  |  |
| 23 | I show more interest in people without disabilities than in people with disabilities | Participatory de novo item | Direct |  |  |  |
| 24 | The provision of specialist staff and support programmes for people with disabilities is currently adequate | Participatory de novo item | Direct |  |  |  |
| 25 | A shift in societal attitudes is necessary to ensure that people with disabilities have a comparable level of opportunities | Participatory de novo item | Reverse |  |  |  |

**Note.** Rows are aligned by item number for compact presentation and do not imply one-to-one retention; the conceptual links are shown in Panel B. 'Direct' and 'Reverse' refer to scoring direction for ableism.

**Supplementary Table 3B. Conceptual mapping from the 25-item Phase-2 pool to the 21-item Phase-3 pre-final pool**

| **Phase-2 source item(s)** | **Phase-3 item(s)** | **Type / provenance of change** | **Rationale** |
| --- | --- | --- | --- |
| Items 1-5, 7, 10, 15, 17 | Item 8 | Concepts consolidated and rewritten | Reduce repetitive merit/success statements and focus on a direct disability-competence/success attribution. |
| Item 6 | Item 9 | Linguistically revised | Retain denial of discrimination while using a concise first-person formulation. |
| Item 8 | Item 13 | Linguistically revised | Retain perceived societal burden and phrase it explicitly as the respondent's opinion. |
| Item 9 | Item 15 | Conceptually softened and revised | Replace the extreme wording 'stay hidden' with a less overt social-segregation statement suitable for detecting subtler attitudes. |
| Items 11, 15, 17, 21 | Item 12 | Conceptually synthesized | Assess justification of unequal opportunities directly and reduce overlap among opportunity-related statements. |
| Item 16 | Item 7 | Conceptually revised | Shift from self-determination wording to equal interpersonal treatment. |
| Item 18 | Item 20 | Reframed and reverse-scored | Express labour-market inclusion positively and as a personal attitude. |
| Item 19 | Item 19 | Linguistically revised | Retain representation content with shorter wording. |
| Item 20 | Item 16 | Linguistically revised | Retain perceived adequacy of medical care in a concise formulation. |
| Item 22 | Item 3 | Perspective shifted | Move from an external statement about pity to self-reported affect. |
| Item 23 | Item 6 | Behavioural focus strengthened | Measure contact avoidance directly rather than relative interest. |
| No direct Phase-2 predecessor | Items 1, 2, 4, 5, 10, 11, 14, 17, 18, and 21 | Newly formulated after Phase 2 | Extend coverage of uncertainty, discomfort, essentialist and benevolent stereotypes, competence, leadership, media visibility, and infantilizing communication identified through qualitative and expert input. |
| Items 12, 13, 14, 24, 25 | No direct Phase-3 successor | Not carried forward as standalone concepts | Content was redundant, strongly normative, or sufficiently represented by the revised pool. |

**Note.** Phase-2 Items 1-13 were translated from the original English SAS; Items 14-25 were de novo items developed through the participatory expert-panel process. All literal Phase-2 wordings were retired, even where their concepts informed a Phase-3 item.

**Supplementary Table 4. Descriptive statistics of the 14 SAS-G items retained after item screening in the Phase-3 student sample (N = 173)**

| **Item** | **Item wording** | **Mean (SD)** | **Range** | **Floor (%)** | **Ceiling (%)** |
| --- | --- | --- | --- | --- | --- |
| 1 | I am often unsure how to behave when I interact with people with disabilities. | 2.5 (1.0) | 1-5 | 13.6 | 0.6 |
| 2 | I often feel uneasy when talking to people with disabilities. | 2.2 (0.9) | 1-5 | 20.2 | 9.8 |
| 3 | I sometimes feel pity for people with disabilities. | 3.3 (1.0) | 1-5 | 5.8 | 6.4 |
| 4 | I believe that a disability also determines the character of a person with disabilities. | 2.5 (1.1) | 1-5 | 21.4 | 1.7 |
| 5 | I think people with disabilities, such as dwarfism or Down syndrome, are cute. | 1.9 (1.0) | 1-5 | 43.9 | 1.7 |
| 6 | I tend to avoid contact with people with disabilities. | 1.7 (0.8) | 1-5 | 44.5 | 0.6 |
| 9 | I believe that discrimination against people with disabilities is no longer a problem in Germany. | 1.7 (0.7) | 1-4 | 42.2 | 0.0 |
| 10 | I think people with disabilities are less competent in many areas. | 2.3 (1.0) | 1-5 | 25.4 | 1.5 |
| 11 | I think social structures make life more difficult for people with disabilities^r^. | 1.8 (0.7) | 1-4 | 34.7 | 0.0 |
| 12 | I think it is justified that people with disabilities do not have the same opportunities as people without disabilities because of their disability. | 1.9 (1.0) | 1-5 | 38.7 | 1.7 |
| 16 | I consider medical care for people with disabilities to be adequate. | 2.5 (0.8) | 1-4 | 12.7 | 0.0 |
| 19 | I think people with disabilities are already sufficiently represented in all areas of society. | 2.0 (0.9) | 1-5 | 34.7 | 0.6 |
| 20 | I think people with disabilities should have greater access to the regular labor market^r^. | 1.9 (0.8) | 1-4 | 31.8 | 0.0 |
| 21 | I tend to use a childish, caring tone when talking to people with disabilities. | 2.2 (1.0) | 1-4 | 30.1 | 0.0 |

**Note.** Response options ranged from 1 = strongly disagree to 5 = strongly agree. ^r^ Reverse-coded. SD, standard deviation.

**Supplementary Table 5.Descriptive statistics of the 21-item pre-final SAS-G in the Phase-3 student sample (N = 173)**

| **Item** | **Item wording** | **Mean (SD)** | **Floor (%)** | **Ceiling (%)** |
| --- | --- | --- | --- | --- |
| 1 | I am often unsure how to behave when I interact with people with disabilities. | 2.5 (1.0) | 13.6 | 0.6 |
| 2 | I often feel uneasy when talking to people with disabilities. | 2.2 (0.9) | 20.2 | 9.8 |
| 3 | I sometimes feel pity for people with disabilities. | 3.3 (1.0) | 5.8 | 6.4 |
| 4 | I believe that a disability also determines the character of a person with disabilities. | 2.5 (1.1) | 21.4 | 1.7 |
| 5 | I think people with disabilities, such as dwarfism or Down syndrome, are cute. | 1.9 (1.0) | 43.9 | 1.7 |
| 6 | I tend to avoid contact with people with disabilities. | 1.7 (0.8) | 44.5 | 0.6 |
| 7 | I think all people with disabilities should be treated as equals^r^. | 1.4 (0.6) | 70.5 | 0.0 |
| 8 | I think people with disabilities cannot perform well and consequently cannot be successful because of their disability. | 1.6 (0.8) | 56.6 | 0.0 |
| 9 | I believe that discrimination against people with disabilities is no longer a problem in Germany. | 1.7 (0.7) | 42.2 | 0.0 |
| 10 | I think people with disabilities are less competent in many areas. | 2.3 (1.0) | 25.4 | 1.5 |
| 11 | I think social structures make life more difficult for people with disabilities^r^. | 1.8 (0.7) | 34.7 | 0.0 |
| 12 | I think it is justified that people with disabilities do not have the same opportunities as people without disabilities because of their disability. | 1.9 (1.0) | 38.7 | 1.7 |
| 13 | I think people with disabilities demand too much from the rest of society. | 1.6 (0.7) | 50.9 | 0.0 |
| 14 | I think people with disabilities should not hold leadership positions. | 1.7 (0.8) | 51.4 | 0.0 |
| 15 | I think people with disabilities should stay among themselves. | 1.2 (0.4) | 82.1 | 0.0 |
| 16 | I consider medical care for people with disabilities to be adequate. | 2.5 (0.8) | 12.7 | 0.0 |
| 17 | I think people with disabilities should have the same political freedoms as people without disabilities^r^. | 1.7 (0.9) | 58.4 | 1.2 |
| 18 | I find it unpleasant to see people with disabilities on television. | 1.4 (0.7) | 71.1 | 0.0 |
| 19 | I think people with disabilities are already sufficiently represented in all areas of society. | 2.0 (0.9) | 34.7 | 0.6 |
| 20 | I think people with disabilities should have greater access to the regular labor market^r^. | 1.9 (0.8) | 31.8 | 0.0 |
| 21 | I tend to use a childish, caring tone when talking to people with disabilities. | 2.2 (1.0) | 30.1 | 0.0 |

**Note.** Items with floor or ceiling effects >50% (Items 7, 8, 13, 14, 15, 17, and 18) were excluded before factor analysis. Response options ranged from 1 = strongly disagree to 5 = strongly agree. ^r^ Reverse-coded. SD, standard deviation.

**Supplementary Table 6.German Symbolic Ableism Scale (SAS-G): final German 10-item set derived after exploratory factor analysis**

| **Item** | **German item wording** | **Subscale** | **Reverse-coded** |
| --- | --- | --- | --- |
| 1 | Ich weiß nicht, wie ich mich verhalten soll, wenn ich mit Menschen mit Behinderungen in Kontakt trete. | Interpersonal Comfort | No |
| 2 | Ich fühle mich im Gespräch mit Menschen mit Behinderungen häufig unwohl. | Interpersonal Comfort | No |
| 6 | Ich versuche, den Kontakt mit Menschen mit Behinderungen zu vermeiden. | Interpersonal Comfort | No |
| 9 | Ich bin der Meinung, dass die Diskriminierung von Menschen mit Behinderungen in Deutschland kein Problem mehr ist. | Denial of Rights and Structural Barriers | No |
| 10 | Ich denke, Menschen mit Behinderungen sind in vielen Bereichen weniger kompetent. | Denial of Rights and Structural Barriers | No |
| 11 | Ich denke, gesellschaftliche Strukturen erschweren das Leben von Menschen mit Behinderungen. | Denial of Rights and Structural Barriers | Yes |
| 16 | Ich halte die derzeitige medizinische Versorgung für Menschen mit Behinderungen für ausreichend. | Denial of Rights and Structural Barriers | No |
| 19 | Ich finde, Menschen mit Behinderungen sind bereits in allen gesellschaftlichen Bereichen ausreichend vertreten. | Denial of Rights and Structural Barriers | No |
| 20 | Ich finde, Menschen mit Behinderungen sollten stärker auf dem regulären Arbeitsmarkt integriert werden. | Denial of Rights and Structural Barriers | Yes |
| 21 | Ich tendiere dazu, im Gespräch mit Menschen mit Behinderungen in einen kindlichen, fürsorglichen Ton zu verfallen. | Interpersonal Comfort | No |

**Note.** Instructions: Please indicate how much you agree with each statement. Responses were recorded on a five-point Likert scale with the following German response options: 1 = stimme überhaupt nicht zu, 2 = stimme eher nicht zu, 3 = weder noch, 4 = stimme eher zu, 5 = stimme voll und ganz zu (corresponding to 1 = strongly disagree and 5 = strongly agree). Items 11 and 20 are reverse-coded and should be recoded before computing subscale means. Higher subscale mean scores indicate more pronounced ableist attitudes. This study provides initial exploratory psychometric evidence; independent confirmatory validation is required.

**Supplementary Table 7.English-language pre-final 21-item version of the SAS-G used before factor analysis**

| **Item** | **English item wording** |
| --- | --- |
| 1 | I am often unsure how to behave when I interact with people with disabilities. |
| 2 | I often feel uneasy when talking to people with disabilities. |
| 3 | I sometimes feel pity for people with disabilities. |
| 4 | I believe that a disability also determines the character of a person with disabilities. |
| 5 | I think people with disabilities, such as dwarfism or Down syndrome, are cute. |
| 6 | I tend to avoid contact with people with disabilities. |
| 7 | I think all people with disabilities should be treated as equals^r^. |
| 8 | I think people with disabilities cannot perform well and consequently cannot be successful because of their disability. |
| 9 | I believe that discrimination against people with disabilities is no longer a problem in Germany. |
| 10 | I think people with disabilities are less competent in many areas. |
| 11 | I think social structures make life more difficult for people with disabilities^r^. |
| 12 | I think it is justified that people with disabilities do not have the same opportunities as people without disabilities because of their disability. |
| 13 | I think people with disabilities demand too much from the rest of society. |
| 14 | I think people with disabilities should not hold leadership positions. |
| 15 | I think people with disabilities should stay among themselves. |
| 16 | I consider medical care for people with disabilities to be adequate. |
| 17 | I think people with disabilities should have the same political freedoms as people without disabilities^r^. |
| 18 | I find it unpleasant to see people with disabilities on television. |
| 19 | I think people with disabilities are already sufficiently represented in all areas of society. |
| 20 | I think people with disabilities should have greater access to the regular labor market^r^. |
| 21 | I tend to use a childish, caring tone when talking to people with disabilities. |

**Note.** Response options ranged from 1 = strongly disagree to 5 = strongly agree. ^r^ Reverse-coded. The German wording is the target-language instrument; the English wording is supplied for transparency and was not independently validated.

**Supplementary Table 8.English-language final 10-item version of the SAS-G after exploratory factor analysis**

| **Item** | **English item wording** |
| --- | --- |
| 1 | I am often unsure how to behave when I interact with people with disabilities. |
| 2 | I often feel uneasy when talking to people with disabilities. |
| 6 | I tend to avoid contact with people with disabilities. |
| 9 | I believe that discrimination against people with disabilities is no longer a problem in Germany. |
| 10 | I think people with disabilities are less competent in many areas. |
| 11 | I think social structures make life more difficult for people with disabilities^r^. |
| 16 | I consider medical care for people with disabilities to be adequate. |
| 19 | I think people with disabilities are already sufficiently represented in all areas of society. |
| 20 | I think people with disabilities should have greater access to the regular labor market^r^. |
| 21 | I tend to use a childish, caring tone when talking to people with disabilities. |

**Note.** Response options ranged from 1 = strongly disagree to 5 = strongly agree. ^r^ Reverse-coded. The German wording is the target-language instrument; the English wording is supplied for transparency and was not independently validated.

**Supplementary Table 9.English-language interview guide used for participatory item development**

| **Question** | **Interview prompt** |
| --- | --- |
| 1 | Have you ever heard the term 'ableism' before? If yes: How do you interpret this term? If not: Do you think that individuals with disabilities are often categorized or stereotyped based on their abilities? Please provide examples or personal experiences. |
| 2 | What does the term 'disability discrimination' mean to you? |
| 3 | Have you personally experienced prejudice or bias related to a disability or functional limitation? If so, could you describe the nature of this experience? |
| 4 | Do you think that the stereotyping or categorization of people with disabilities in Germany needs to be addressed? Please explain your perspective. |
| 5 | Do you perceive people with disabilities as being inherently different from those without disabilities? Please explain your perspective. |
| 6 | Do you believe that people with disabilities should have greater visibility in politics, the media, and public events? Please explain your perspective. |
| 7 | In your opinion, what factors contribute to the categorization of individuals with disabilities? |
| 8 | Do you think that public institutions, healthcare facilities, cafés, and other public spaces should take additional measures to enhance accessibility? Please explain your perspective. |
| 9 | Do you think that people with disabilities should experience greater inclusion in everyday educational settings (e.g., schools and universities) and in the mainstream labor market? Please explain your perspective. |

**Note.** This guide was used flexibly in Phase 1 to encourage open discussion and elaboration.

**Supplementary Table 10.Reasons for item exclusion in Phase 3**

| **Item** | **Reason for exclusion** | **Relevant finding** |
| --- | --- | --- |
| 7 | Pronounced floor effect | 70.5% of responses in the lowest category |
| 8 | Pronounced floor effect | 56.6% in the lowest category |
| 13 | Pronounced floor effect | 50.9% in the lowest category |
| 14 | Pronounced floor effect | 51.4% in the lowest category |
| 15 | Pronounced floor effect | 82.1% in the lowest category |
| 17 | Pronounced floor effect | 58.4% in the lowest category |
| 18 | Pronounced floor effect | 71.1% in the lowest category |
| 4 | Insufficient factor loading | No factor loading >= .32 in the initial EFA |
| 3 | Loading on a separate third factor | Loading .555 on Factor 3; the factor contained only Items 3 and 5 |
| 5 | Loading on a separate third factor | Loading .524 on Factor 3; the factor contained only Items 3 and 5 |
| 12 | Cross-loading | Primary loading .442; secondary loading .326 in the two-factor solution |

**Note.** Endpoint concentration was treated as insufficient variability for the present factor analysis; it does not establish that the underlying content is unimportant. Social desirability or strong normative rejection may contribute to these distributions.

**Supplementary Table 11.Item-level internal-consistency statistics for the final SAS-G**

| **Factor** | **Item** | **Corrected item-total correlation** | **Cronbach's alpha if item deleted** |
| --- | --- | --- | --- |
| Denial of Rights and Structural Barriers | 9 | .635 | .753 |
| Denial of Rights and Structural Barriers | 10 | .451 | .802 |
| Denial of Rights and Structural Barriers | 11 | .426 | .794 |
| Denial of Rights and Structural Barriers | 16 | .604 | .756 |
| Denial of Rights and Structural Barriers | 19 | .624 | .751 |
| Denial of Rights and Structural Barriers | 20 | .642 | .749 |
| Interpersonal Comfort | 1 | .541 | .577 |
| Interpersonal Comfort | 2 | .623 | .529 |
| Interpersonal Comfort | 6 | .492 | .620 |
| Interpersonal Comfort | 21 | .285 | .747 |

**Note.** Scale-level estimates: Denial of Rights and Structural Barriers, alpha = .81 and omega = .83 (6 items); Interpersonal Comfort, alpha = .70 and omega = .74 (4 items).
